# Metagenomic Analysis of the Microbial Diversity in Solid Waste from Okhla Landfill, New Delhi, India

**DOI:** 10.1128/MRA.00921-20

**Published:** 2020-11-12

**Authors:** Bindu Yadav, Atul Kumar Johri, Meenakshi Dua

**Affiliations:** a Microbial Ecology Laboratory, School of Environmental Sciences, Jawaharlal Nehru University, New Delhi, India; b School of Life Sciences, Jawaharlal Nehru University, New Delhi, India; Indiana University, Bloomington

## Abstract

The Okhla landfill site is consistently in the news for having pollution levels higher than the city average. Here, we report the taxonomic characterization of the microbial diversity of Okhla landfill solid waste. The metagenome analyses revealed the microbial and metabolic diversity of the site.

## Abstract (precis)

The Okhla landfill site is consistently in the news for pollution levels higher than the city average. Here, we report the taxonomic and functional characterization of the microbial diversity of Okhla landfill solid waste. The metagenome analyses revealed the microbial and metabolic diversity of the site.

## ANNOUNCEMENT

Over 10,050 tons of municipal solid waste (MSW) is generated in Delhi every day, and only 50% of this waste is treated (https://www.downtoearth.org.in/news/waste/why-a-landfill-and-waste-to-energy-plant-in-delhi-ridge-is-a-bad-idea-61191). The Okhla landfill site has been filled much beyond its commissioned capacity and is spread over 32 acres in the heart of the city, a stone’s throw away from residential areas. Often, the contaminants in leachate can be linked to household hazardous waste, apart from industrial waste (1). The microbial communities present in the soil can transform most pollutants and organic matter into less toxic compounds and convert waste material into mineralized end products like water, CO_2_, and CH_4_ (2). High-throughput culture-independent techniques like metagenomics provide a holistic view of the structural and functional diversity of the microbes in landfill soil (3). We report the phylogenetic characterization of the microbiota in solid waste from the Okhla landfill.

Samples were collected in November 2017 (daytime average temperature, 19°C; humidity, 73%) from the Okhla landfill site (28.5626N, 77.2914E). All the samples were crushed, evenly mixed, and sieved through 0.2-mm mesh to prepare a composite sample. Genomic DNA was extracted using the NucleoSpin soil kit (TaKaRa Bio, USA). Paired-end (PE) sequencing libraries were prepared using the Illumina TruSeq Nano DNA library prep kit. The size-selected product was PCR amplified, and the enriched libraries were analyzed on a 4200 TapeStation system (Agilent Technologies). PE Illumina libraries with a mean fragment size of 532 bp were sequenced on a NextSeq 500 instrument using 2 × 150-bp chemistry. The raw PE reads were processed using Trimmomatic v0.38 to obtain 15,683,291 high-quality (HQ) reads. The HQ reads were assembled into scaffolds using CLC Genomics Workbench v9.5.2. Genes were predicted from the assembled scaffolds using Prodigal v2.6.3 with default parameters. Taxonomic analysis of predicted genes was carried out using Kaiju v1.6.2. Functional analysis to provide gene annotations through Clusters of Orthologous Groups (COG) and KEGG was carried out using Cognizer v0.9b. Default parameters were used for all software except where otherwise noted.

Of the 139,123 assembled scaffolds, 300,708 genes were predicted, with an average gene length of 550 bp. Approximately 21% of the taxonomic analyses comprised unidentified reads. The 78.84% of identified reads revealed 78.29% cellular organisms and 0.55% viruses. *Bacteria* (77.83%) dominated the microbial diversity, while *Eukaryota* (0.40%) formed a very small fraction, and *Archaea* (<0.06%) were almost negligible. The most abundant phylum among *Bacteria* was *Actinobacteria* (48.5%), represented strongly by *Streptomyces* and *Saccharomonospora.* The other phyla, in order of abundance, were *Proteobacteria*, *Firmicutes*, *Bacteroidetes*, *Planctomycetes*, and *Cyanobacteria*. Functional classification using COG categorized genes as controlling general function (14%), amino acid transport and metabolism (9%), energy production and conversion (6%), carbohydrate transport and metabolism (7.2%), and transcription (7.8%). Ontology analysis based on the KEGG classified genes as controlling metabolism (36%), environmental information processing (13%), cellular processes (3%), and genetic information processing (6%).

Metagenomics is applied to understand the identities, the ecologies, and the functional diversities of microorganisms in landfill sites to devise efficient consortia of microbial communities for bioremediation. The dominance of *Saccharomonospora* is of interest, because the members of this phylum originate from diverse habitats and play a prominent role in the process of composting (4).

### Data availability.

This whole-genome shotgun project has been deposited at DDBJ/ENA/GenBank under the accession number JACYYJ000000000. The version described in this paper is the first version, JACYYJ010000000. The raw metagenome sequence has been uploaded to the NCBI Sequence Read Archive (SRA) under the accession number SRR11086757. The BioSample accession number is SAMN14103086.
